# Computed tomography radiomics identification of T1–2 and T3–4 stages of esophageal squamous cell carcinoma: two-dimensional or three-dimensional?

**DOI:** 10.1007/s00261-023-04070-1

**Published:** 2023-10-16

**Authors:** Yang Li, Li Yang, Xiaolong Gu, Qi Wang, Gaofeng Shi, Andu Zhang, Meng Yue, Mingbo Wang, Jialiang Ren

**Affiliations:** 1 Department of Radiology, The Fourth Hospital of Hebei Medical University, Shijiazhuang, 050000 Hebei Province People’s Republic of China; 2 Department of Radiotherapy, The Fourth Hospital of Hebei Medical University, Shijiazhuang, 050000 Hebei Province People’s Republic of China; 3 Department of Pathology, The Fourth Hospital of Hebei Medical University, Shijiazhuang, 050000 Hebei Province People’s Republic of China; 4 Department of Thoracic Surgery, The Fourth Hospital of Hebei Medical University, Shijiazhuang, 050000 Hebei Province People’s Republic of China; 5 GE Healthcare China, Beijing, 100176 People’s Republic of China

**Keywords:** Esophageal squamous cell carcinoma, Tumor stage, Computed tomography, Radiomics

## Abstract

**Background:**

To evaluate two-dimensional (2D) and three-dimensional (3D) computed tomography (CT) radiomics analysis for the T stage of esophageal squamous cell carcinoma (ESCC).

**Methods:**

398 patients with pathologically confirmed ESCC were divided into training and testing sets. All patients underwent chest CT scans preoperatively. For each tumor, based on CT images, a 2D region of interest (ROI) was outlined on the largest cross-sectional area, and a 3D ROI was outlined layer by layer on each section of the tumor. The radiomics platform was used for feature extraction. For feature selection, stepwise logistic regression was used. The receiver operating characteristic (ROC) curve was used to assess the diagnostic performance of the 2D radiomics model versus the 3D radiomics model. The differences were compared using the DeLong test. The value of the clinical utility of the two radiomics models was evaluated.

**Results:**

1595 radiomics features were extracted. After screening, two radiomics models were constructed. In the training set, the difference between the area under the curve (AUC) of the 2D radiomics model (AUC = 0.831) and the 3D radiomics model (AUC = 0.830) was not statistically significant (p = 0.973). In the testing set, the difference between the AUC of the 2D radiomics model (AUC = 0.807) and the 3D radiomics model (AUC = 0.797) was also not statistically significant (p = 0.748). A 2D model was equally useful as a 3D model in clinical situations.

**Conclusion:**

The performance of 2D radiomics model is comparable to that of 3D radiomics model in distinguishing between the T1-2 and T3-4 stages of ESCC. In addition, 2D radiomics model may be a more feasible option due to the shorter time required for segmenting the ROI.

## Introduction

Esophageal cancer (EC) had been identified as the sixth leading cause of cancer-related mortality, posing a substantial challenge to global public health [1]. Globally, esophageal squamous cell carcinoma (ESCC) was the most common histologic subtype [2]. The mortality rate among EC patients ranged from 15 to 20% [3]. The tumor-node-metastasis (TNM) system was the most widely used staging system in clinical practice [4]. Oncology hospitals and medical centers had widely adopted it as the primary approach for cancer clinical, pathological, and imaging reporting. Multidisciplinary treatment approaches could improve outcomes and prognosis for EC patients, especially those at advanced tumor stages. Tumors at different stages could be treated with different strategies, so accurate staging is crucial in guiding treatment decisions [5].

Currently, EC could be treated in a variety of ways [6]. Accurate T-stage helps to develop the treatment plan for EC. For the T1/T2 stage of EC, surgery is the treatment of choice, while for the T3/T4a stage, a combination of neoadjuvant chemotherapy and surgery is recommended [7]. Endoscopic ultrasound (EUS) is an operator-dependent modality highly influenced by the operator’s experience and subjective proficiency, so the result could be affected. Tumor-associated edema can lead to overstaging, while limited tumor penetration might lead to understaging. For patients with a noticeable narrowing of the esophageal lumen, if endoscopy could not pass through the lesion, it might be impossible to accurately evaluate the whole tumor [8]. Computer tomography is a non-invasive imaging method for determining the stage of EC [9]. However, the accuracy for stages T1/T2 was lower than that of EUS [10–12]. Dynamic contrast-enhanced multi-slice computed tomography (CT) had been shown to improve the accuracy of the T stage of EC [13]. PET/CT could not accurately identify the depth of tumor infiltration, limiting its value in assessing the T stage of EC [14, 15]. In a previous study, MRI was found to be unsatisfactory for the T stage of EC, especially for early-stage tumors [16]. With the development of MRI imaging techniques, the diagnostic performance of MRI for tumor staging had improved compared to CT and EUS for resectable EC [13, 17, 18]. However, MRI also had some disadvantages that were difficult to overcome, such as its high cost, long examination time, and inability to analyze images due to patient breathing and motion artifacts. In addition, MRI had several contraindications. For example, patients with pacemakers, coil implants, or generalized anxiety disorders were not candidates for MRI. Radiomics could extract high-throughput image features from radiological images, providing a wealth of additional information hidden behind the images [19]. Currently, CT-based radiomics had shown promising applications in digestive system tumors [20, 21]. It had been demonstrated by Liu et al. [22] that texture analysis of CT images could be used to assess the preoperative staging of ESCC. Wu et al. [23] developed a radiomics model for preoperative identification of stages I–II and III–IV ESCC. Yang et al. [20] revealed that CT-based radiomics could predict the pathological T stage of patients with ESCC preoperatively, which, in combination with existing examinations, could help in the accurate assessment of patients. Therefore, tumor segmentation was a key step in many radiomics-related studies [24–26].

There had been a long debate about whether to use a two-dimensional (2D) or three-dimensional (3D) region of interest (ROI) to perform the radiomics analysis [25, 27, 28]. Therefore, our study aimed to analyze 2D and 3D models for predicting the T stage of ESCC in order to provide a method for the pre-treatment evaluation of ESCC.

## Methods

### Patient population and study design

**Fig. 1:**
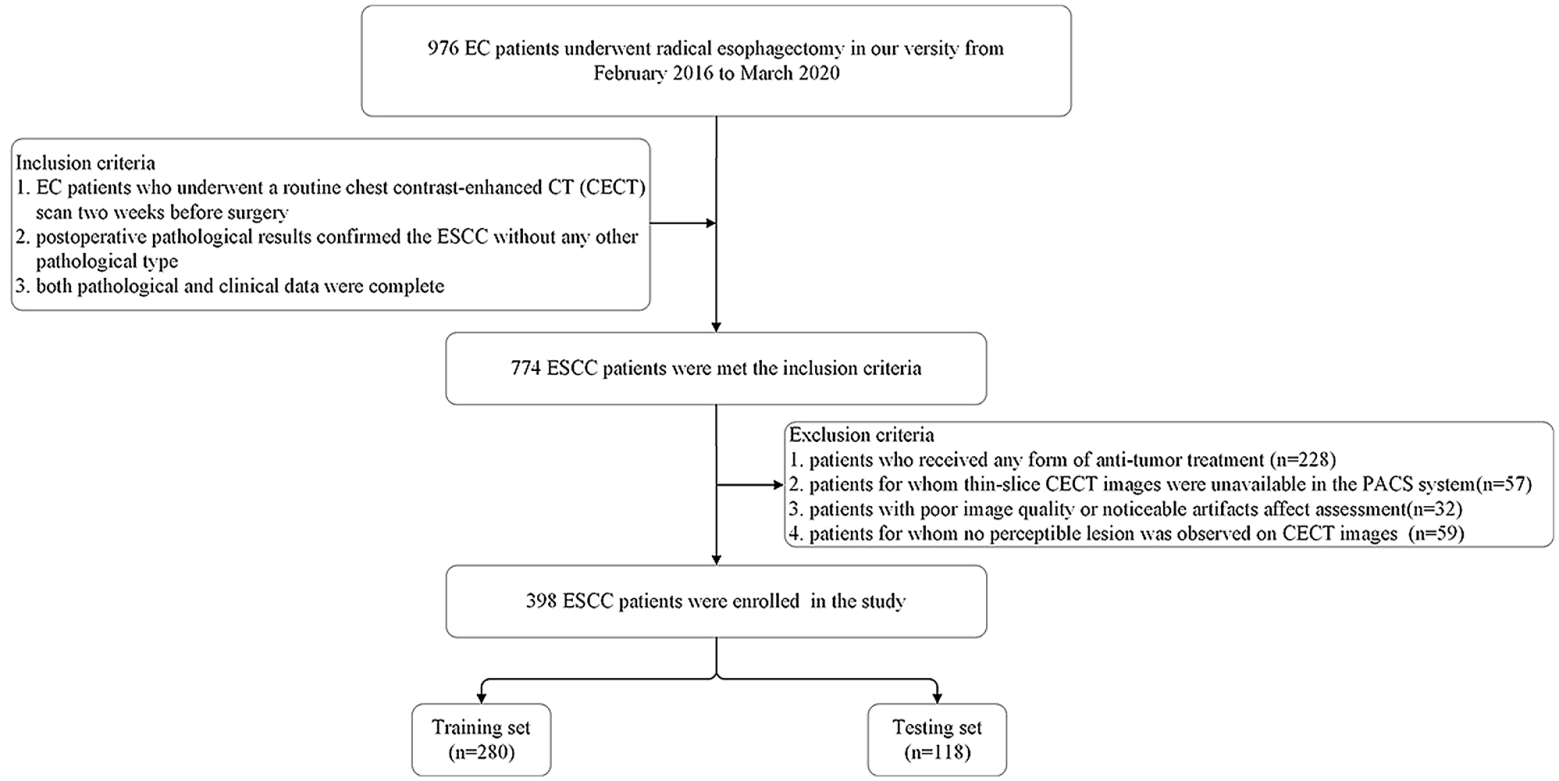
Flowchart illustrating the included patients

A retrospective study was conducted on patients who underwent radical esophagectomy at the Fourth Hospital of Hebei Medical University from February 2016 to March 2020. The inclusion criteria were: (1) EC patients who underwent a routine chest contrast-enhanced CT (CECT) scan two weeks before surgery; (2) postoperative pathological results confirmed the ESCC without any other pathological type; and (3) both pathological and clinical data were complete. The exclusion criteria were: (1) patients who received any form of anti-tumor treatment (n = 228); (2) patients for whom thin-slice CECT images were unavailable in the PACS system (n = 57); (3) patients with poor image quality or noticeable artifacts affect assessment(n = 32); (4) patients for whom no perceptible lesion was observed on CECT images (n = 59). Finally, 398 ESCC patients were included and were randomly divided into a training set and a testing set at a ratio of 7:3. A flowchart of the patient selection process is shown in Fig. 1.

### Clinical and pathological characteristics collection

A retrospective analysis of the postoperative histopathological characteristics of the patients was conducted, including T stage (divided into T1-2 and T3-4), lymph node status (positive or negative), length, thickness, histological grade, lymphovascular invasion (LVI), perineural invasion (PNI), and pTNM stage. According to the 8th Ed. of the AJCC/UICC staging system, the staging had been reclassified.

### Image acquisition and tumor segmentation

The images were obtained from our hospital's PACS system and imported into 3D Slicer software for tumor segmentation. All images were acquired using two commercial CT scanners. Scanner 1: a 128-slice second-generation dual-source CT scanner (SOMATOM Definition Flash, Siemens Healthcare, Forchheim, Germany). Scanner 2: a 256-slice CT scanner (Revolution CT, GE Healthcare, Milwaukee, USA). The tube voltage was 120 kVp, the tube current was set to auto mAs, the slice thickness was 5.0 mm with increments of 5.0 mm, and the reconstructed thin-slice thickness was 1-2 mm. After intravenous injection of contrast agent (3.0–4.0 ml/s, 1.5 ml/kg, Iohexol,300 mg I/ml) via a syringe pump, an arterial phase scan was performed after a 30 seconds delay, followed by a 20 ml saline flush.

**Fig. 2:**
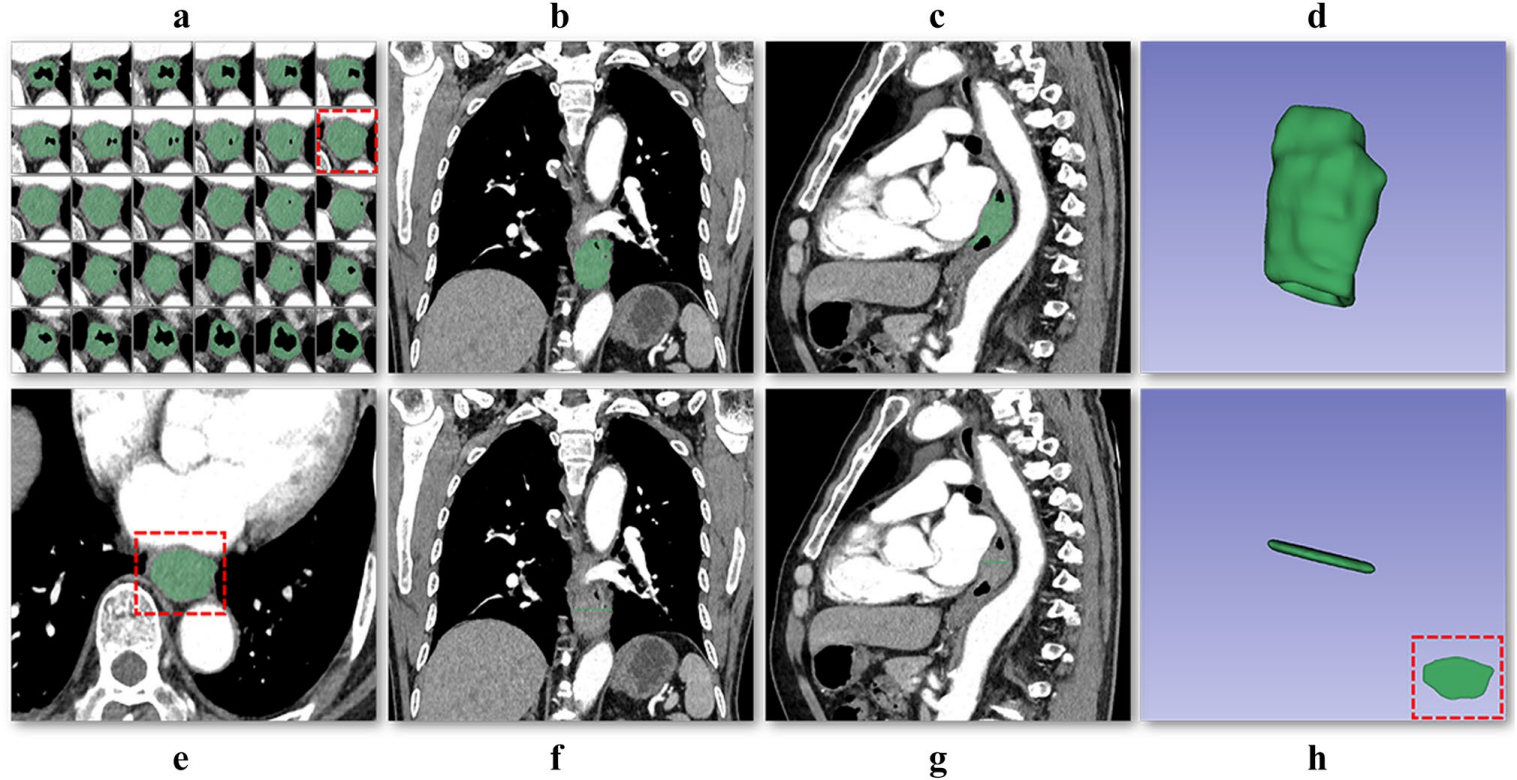
The process of tumor segmentation. The 3D ROI covered the whole tumor area by delineating the tumor tissue layer by layer on axial CT images (**a**). Subsequently, adjustments were made with reference to the coronal (**b**) and sagittal (**c**) CT images to finally reconstruct the 3D ROI (**d**). The 2D ROI was delineated on the largest cross-sectional area of the tumor (**e**). Subsequently, adjustments were made with reference to the coronal (**f**) and sagittal (**g**) CT images to finally reconstruct the 2D ROI (**h**)

Tumor segmentation was performed using mediastinal window settings thin-slice CECT images. The images were linearly interpolated with a voxel size of 1x1x1 mm. The criterion for diagnosing esophageal wall abnormalities was a thickness greater than 5 mm on the transverse axis of the CECT images [29–31]. 2D ROI was delineated on the largest cross-sectional area of the tumor, while 3D ROI was delineated on each slice. The process of tumor segmentation is shown in Fig. 2.

The reliability of feature extraction was assessed by calculating the inter- and intra-class correlation coefficient (ICC). 30 patients were randomly selected, and two radiologists delineated 2D and 3D ROIs for each tumor. One radiologist repeated the segmentation of these 30 tumors one month later. The ICC value was 0.75. If there were potential disagreements, they would be resolved by seeking consensus. During the segmentation process, gas within the esophagus was excluded, and surrounding soft tissues, blood vessels, and bone structures were avoided.

### Radiomics features selection and model construction

2D and 3D ROIs segmentation were performed for each tumor, and feature extraction was performed using the PyRadiomics platform in nii.gz format. The images were discretized in grayscale with bandwidth set to 25. In addition, these features also include images filtered with Gaussian Laplacian (LoG, σ: 1, 3, 5), wavelet, logarithmic, gradient, and local binary pattern (LBP). Moreover, the 2D radiomics features included nine morphological features, while the 3D radiomics features included 14 morphological features. To evaluate the relationship between the two sets, unsupervised clustering and radiomics heat maps were used. 2D and 3D radiomics features were extracted, respectively. All radiomics features were preprocessed with z-score method in the training set and applied in the test set.

**Fig. 3:**
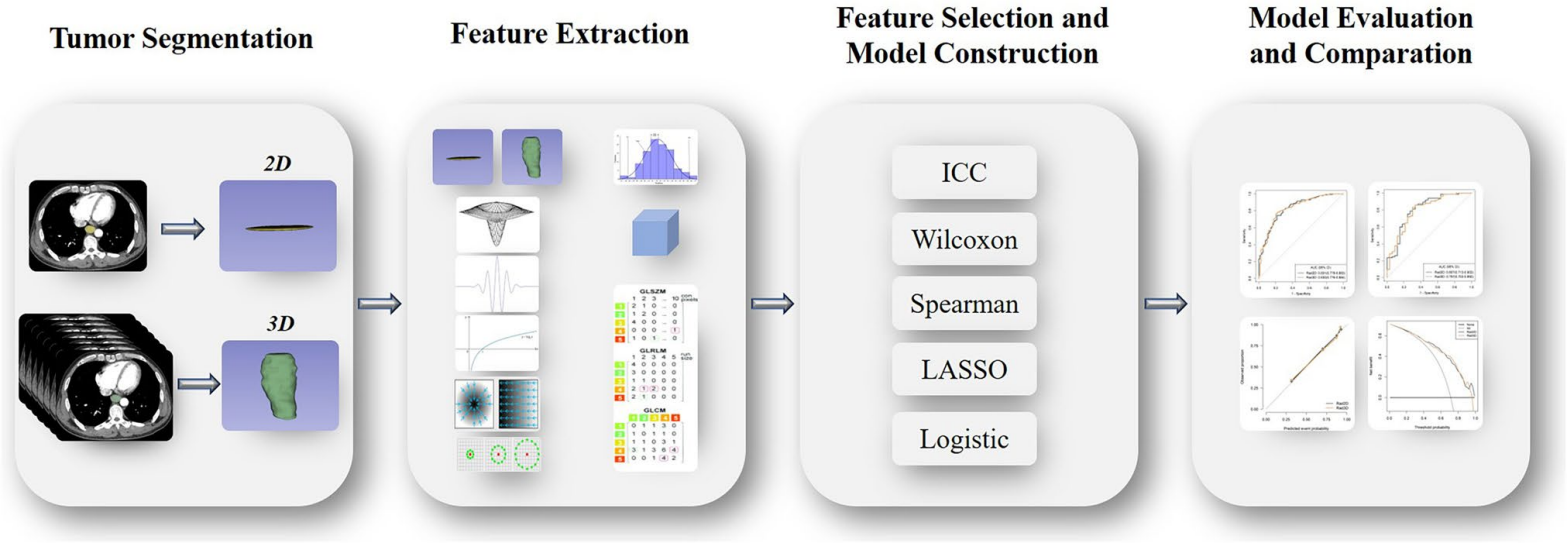
Workflow of radiomics analysis

The process of selecting radiomics features and building models involved five consecutive steps. Firstly, radiomics features extracted with an ICC higher than 0.75 were retained. Secondly, the Wilcoxon rank-sum test was used. Thirdly, Spearman correlation analysis removed features with correlation coefficients greater than 0.9. To select the most useful predictive features in the training set, the least absolute shrinkage and selection operator (LASSO) method was applied. Logistic regression with 10-fold cross-validation was involved. Finally, stepwise logistic regression was used to select the feature set with the minimum Akaike information criterion (AIC) and incorporate it into the logistic regression model. The workflow of radiomics analysis is shown in Fig. 3.

### Statistical analysis

All patients were divided into T1-2 and T3-4 stage groups. Continuous variables with a normal distribution were expressed as mean ± standard deviation, whereas those with a non-normal distribution were described as median (interquartile range). The independent sample t-test was used to compare continuous variables with a normal distribution. The Mann-Whitney U test was used to compare continuous variables with a non-normal distribution. Categorical variables were expressed as counts and compared using a chi-square test. The receiver operating characteristic curve (ROC) and the area under the curve (AUC) were used to evaluate the diagnostic performance of the model. The DeLong test was performed to compare the differences between the 2D and 3D radiomics models. The model's sensitivity, specificity, accuracy, positive predictive value (PPV), and negative predictive value (NPV) were calculated. The Hosmer-Lemeshow (HL) test and calibration curve were used to evaluate the degree of fit of the radiomics model, and decision curve analysis (DCA) was used to evaluate the clinical application value of the model. All tests were two-tailed, and a p-value of less than 0.05 was considered statistically significant. R software (version 4.2.2) was used for all statistical analyses and modeling.

## Results

### Patient clinical and pathological characteristics

Three hundred ninety-eight patients with ESCC, including 266 males and 132 females, were enrolled in this study. The ages of the patients ranged from 37 to 80 years. There were 113 patients with the T1-2 stages, including 30 cases of the T1 stage and 83 cases of the T2 stage, and 285 patients with the T3-4 stages, including 281 cases of T3 and 4 cases of T4a. There was no statistically significant difference between patients with T1-2 and T3-4 stages. However, the two groups had statistically significant differences regarding gender, pLength, pThick, tumor differentiation, lymph node metastasis, PNI, LVI status, and pTNM stage. The detailed clinical and pathological characteristics were provided in Table 1.

**Table 1:** Clinical and pathological characteristics of esophageal squamous cell carcinoma patients

Variable	Training			Testing		
	T1–2 (N = 80)	T3–4 (N = 200)	*P*	T1–2 (N = 33)	T3–4 (N = 85)	*P*
Gender (n)			0.001^a^			1.000^a^
Male	41	145		22	58	
Female	39	55		11	27	
Age (n)			0.953^a^			0.780^a^
≤65 years	49	125		17	48	
>65 years	31	75		16	37	
Location (n)			0.008^a^			0.314^a^
Upper	9	12		0	4	
Middle	44	148		22	61	
Lower	27	40		11	20	
Degree (n)			0.655^a^			0.773^a^
I–II	53	138		22	58	
III	89	62		11	26	
N (n)			0.0851^a^	18	38	0.450^a^
Positive	47	93		15	47	
Negative	33	107				
pTNM (n)			0.007^a^			0.053^a^
I–II	53	95		22	38	
III-IV	27	105		11	47	
LVI (n)			0.182^a^			0.012^a^
Positive	63	140		30	56	
Negative	17	60		3	29	
PNI (n)			0.001^a^			0.003^a^
Positive	71	121		0	52	
Negative	9	79		3	33	
pLength (cm)	3.000 [2.275;3.500]	3.500 [3.000;4.500]	<0.001^b^	3.500 [2.500;4.000]	3.500 [3.000;4.500]	0.071^b^
pThick (cm)	1.000 [0.600;1.000]	1.000 [1.000;1.500]	<0.001^b^	1.000 [0.700;1.300]	1.200 [1.000;1.500]	0.003^b^
CEA (ng/ml)	2.795 [2.105;3.360]	2.925 [2.100;3.360]	0.687^b^	2.920 [1.970;3.360]	2.880 [2.120;3.360]	0.683^b^
SCCA (ng/ml)	1.000 [0.700;1.710]	1.300 [0.780;1.710]	0.052^b^	0.900 [0.700;1.450]	1.200 [0.700;1.710]	0.190^b^

^a^Pearson’s Chi-squared test

^b^Mann-Whitney U test

*LVI* lymphovascular invasion, *PNI* perineural invasion, *CEA* carcinoembryonic antigen, *SCCA* squamous cell carcinoma antigen

### Radiomics features selection and model construction

**Fig. 4:**
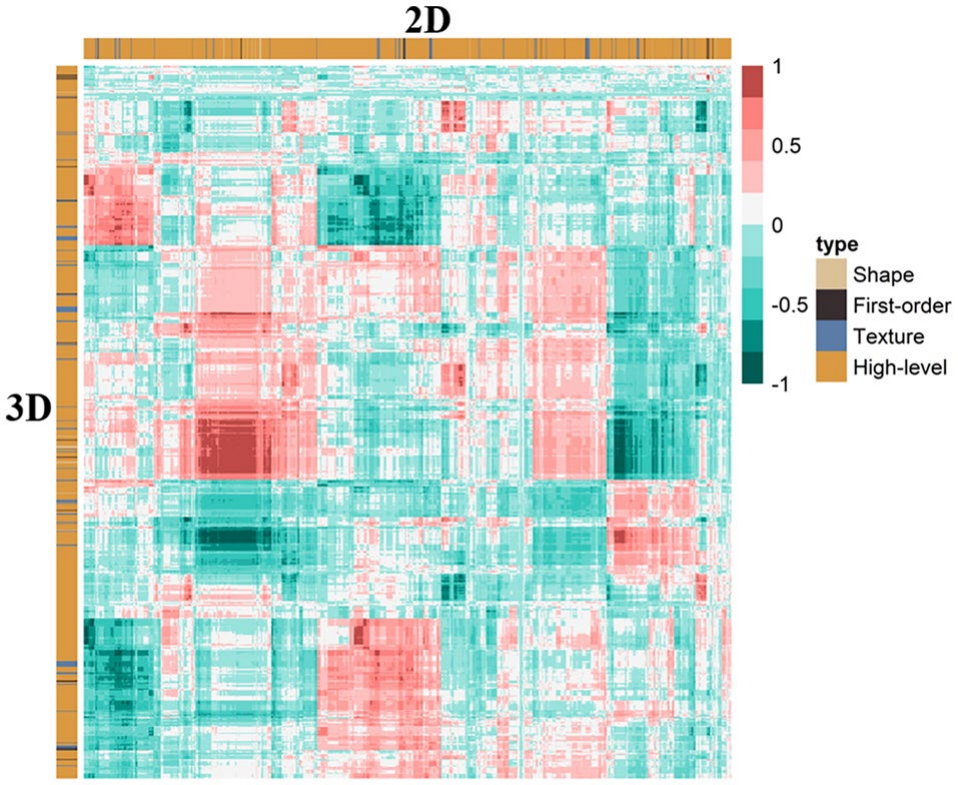
Heat map of radiomics feature cluster containing four distinct classifications. The areas that appear redder indicate stronger correlations between the 2D and 3D radiomics features, while darker green areas signify weaker correlations

**Fig. 5:**
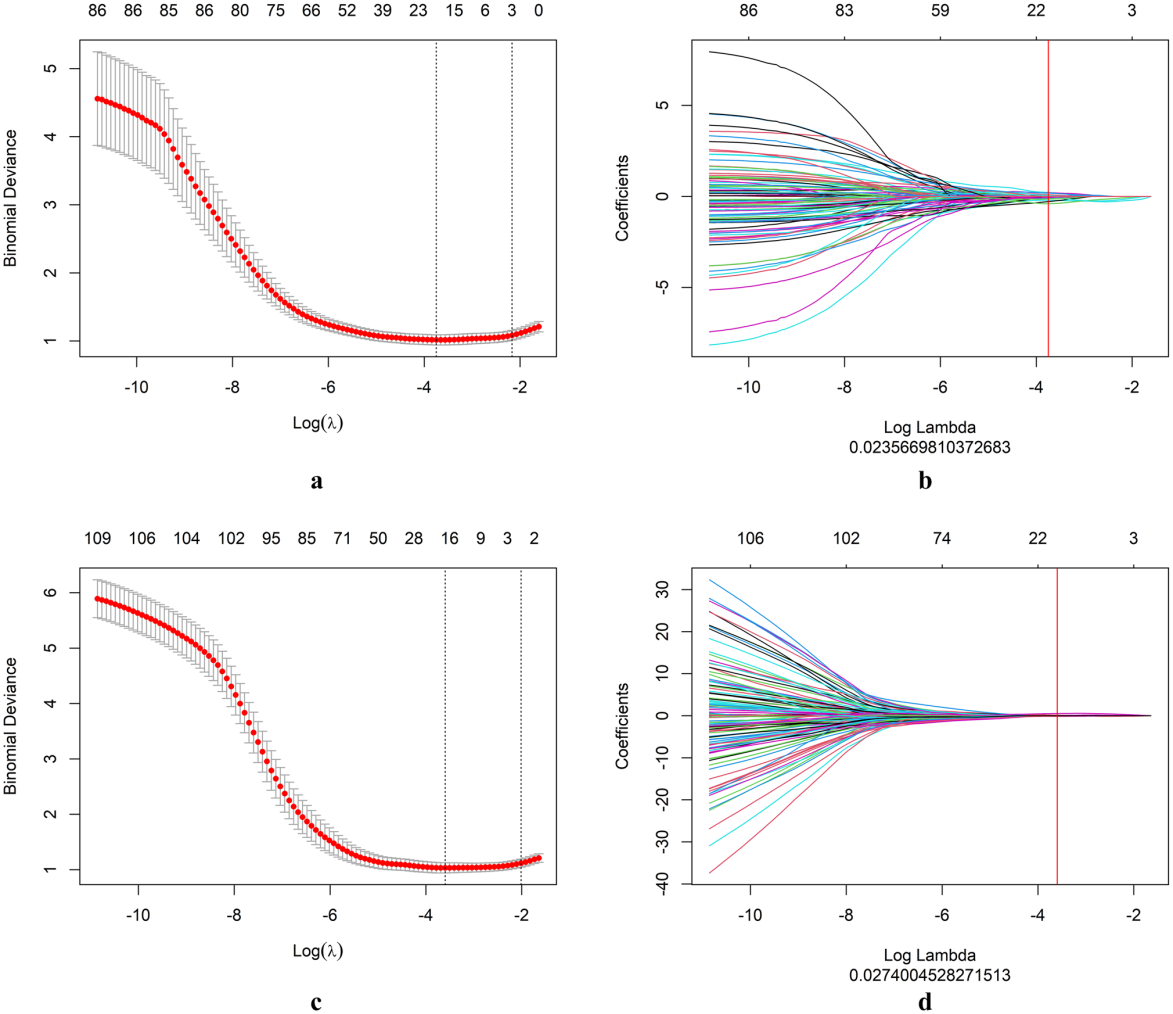
The least absolute shrinkage and selection operator (LASSO) logistic regression for 2D and 3D radiomics feature selection. Specifically, for 2D radiomics, we selected log (λ)  = -− 1.628 and λ = 0.024 (**a**, **b**), and for 3D radiomics, we selected log (λ) = −1.562 and λ = 0.027 (**c**, **d**). We then plotted the LASSO coefficient profile for the 86 and 123 radiomics features in 2D and 3D, respectively. The vertical red line in each plot represents the value of λ selected using 10-fold cross-validation

**Fig. 6:**
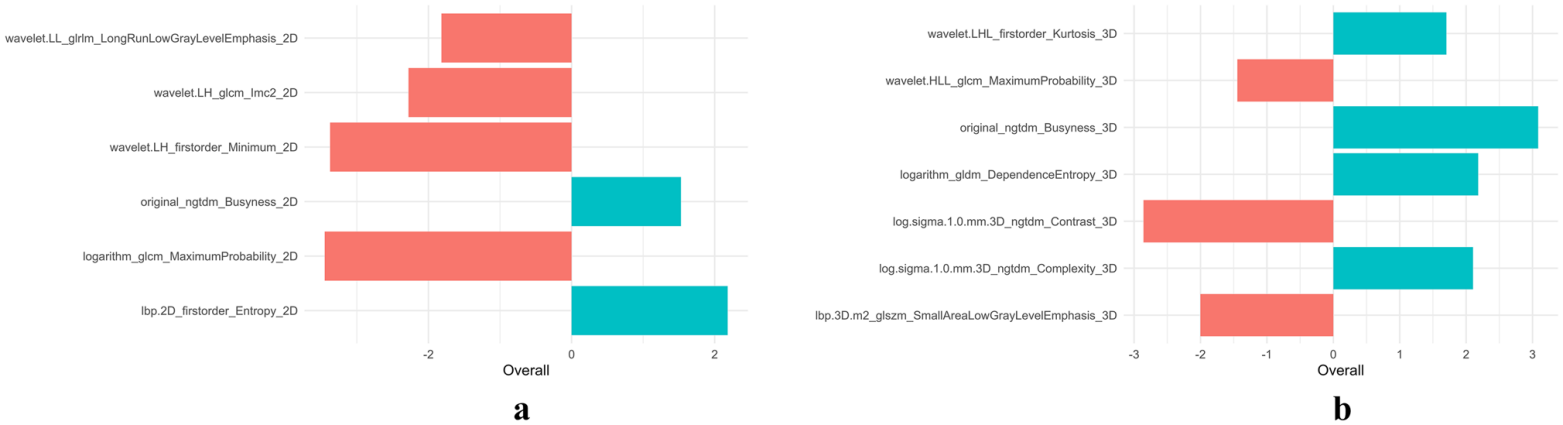
The selected radiomics features and their coefficients in the training set (**a**) and testing set (**b**). The red bars represented negative coefficients, and the light blue bars represented positive coefficients. Negative coefficients were depicted by red bars, while positive coefficients were shown by blue bars. The greater the length of the bars, the greater the absolute value of the coefficients

**Fig. 7:**
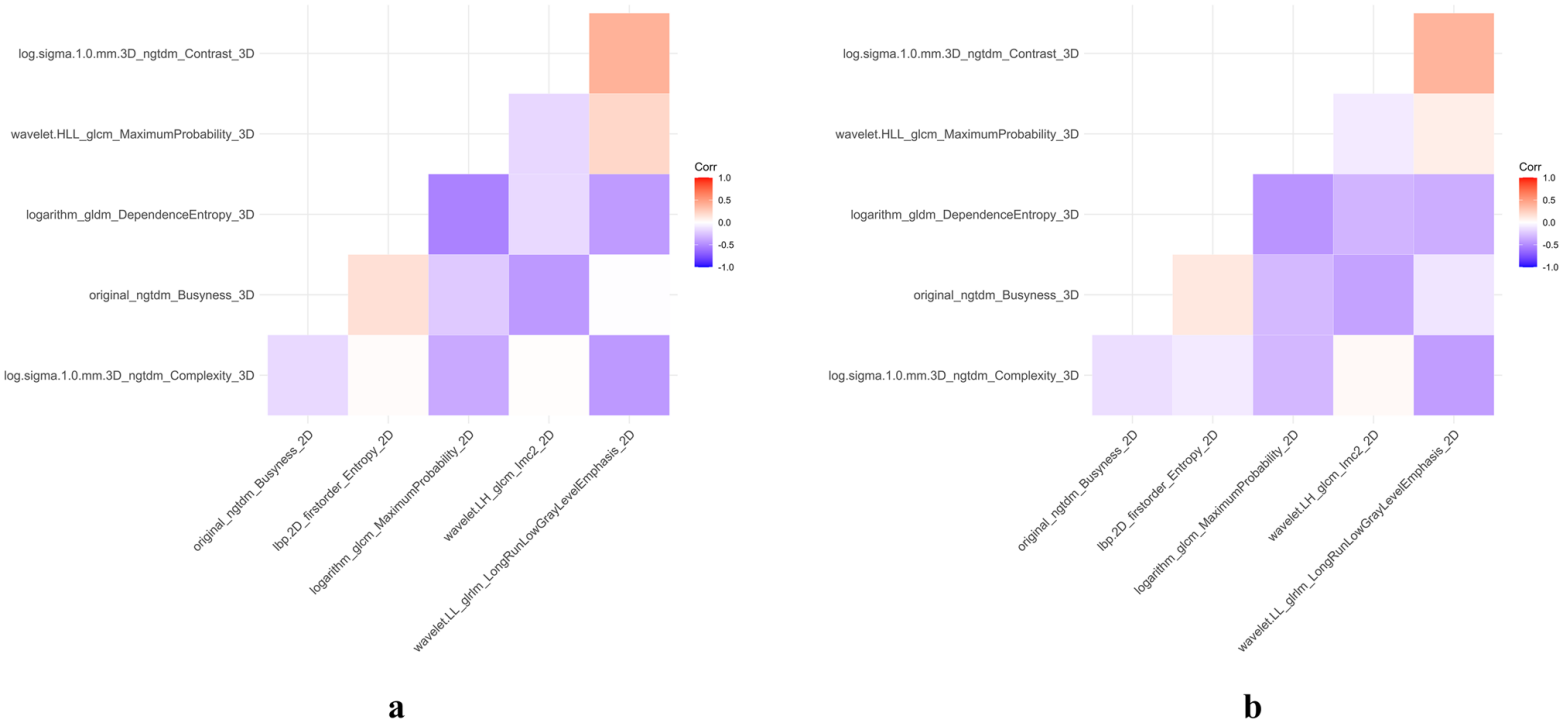
The heat map displays the correlation coefficients between radiomics features in the training set (**a**) and testing set (**b**)

A total of 1595 radiomics features were extracted from the 2D and 3D ROIs of each tumor, respectively. We used unsupervised clustering analysis to visualize the correlation between 2D and 3D radiomics features using a heatmap (Fig. 4). The ICC analysis revealed that 492 2D radiomics features and 1350 3D radiomics features had ICC values greater than 0.05, respectively, indicating good consistency and reproducibility. 431 2D radiomics features and 741 3D radiomics features showed statistically significant differences between patients with T1-2 and T3-4 stage tumors. These radiomics features were included in subsequent Spearman correlation analyses. Radiomics features with an ICC greater than 0.9 were discarded, leaving 86 2D radiomics features and 123 3D radiomics features for LASSO analysis (Fig. 5). The minimum criterion was used to determine the selection of the tuning parameter (λ). After LASSO analysis, 16 radiomics features were retained. Finally, multivariate stepwise logistic regression was used to establish the 2D and 3D radiomics models, respectively, and the feature set with the lowest AIC value was retained. Figure 6 describes the selected 2D and 3D radiomics features and their coefficients. The correlation between the selected 2D and 3D radiomics features was analyzed using Spearman correlation and visualized as a heat map (Fig. 7). For each patient, the radiomics score (Radscore) was calculated, which could be expressed as:

2D Radscore=1.3371+original_ngtdm_Busyness_2D*0.3846-wavelet. LH firstorder_Minimum_2D*0.9596-wavelet. LH glcm_Imc2_2D*0.5414-wavelet. LL glrlm_LongRunLowGrayLevelEmphasis_2D*0.5414-wavelet. LL glrlm_LongRunLowGrayLevelEmphasis_2D*0.3355-logarithm_glcm_MaximumProbability_2D*0.6636+1bp.2D_firstorder_Entropy_2D.

3D Radscore=1.2839+original_ngtdm_Busyness_3D*0.8804+log.sigma.1.0.mm.3D_ngtdm_Complexity_3D*0.5897-log.sigma.1.0.mm.3D_ngtdm_Contrast_3D*0.6159+wavelet.LHL_firstorder_Kurtosis_3D*0.4180-wavelet.HLL_glcm_MaximumProbability_3D*0.274+logarithm_gldm_DependenceEntropy_3D*0.4766-lbp.3D.m2_glszm_SmallAreaLowGrayLevelEmphasis_3D.

**Fig. 8:**
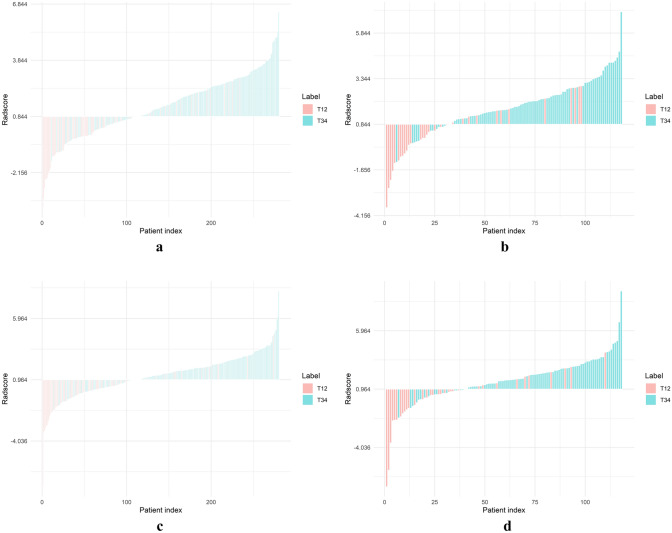
The bar charts displaying the Radscore of 2D (**a**, **b**) and 3D (**c**, **d**) radiomics models in training and testing sets, respectively. The red bars indicated T1–2 stage patients, while the light green bars represented the Radscore of T3–4 stage patients. Correctly identified T1–2 stage patients were shown by red bars below the threshold, and incorrect identifications were represented by red bars above the threshold. Blue bars above the threshold indicated correctly identified T3–4 stage patients, and those below indicated incorrect identifications

The bar charts of 2D and 3D Radscore is shown in Fig. 8.

### Model evaluation and comparison

**Fig. 9:**
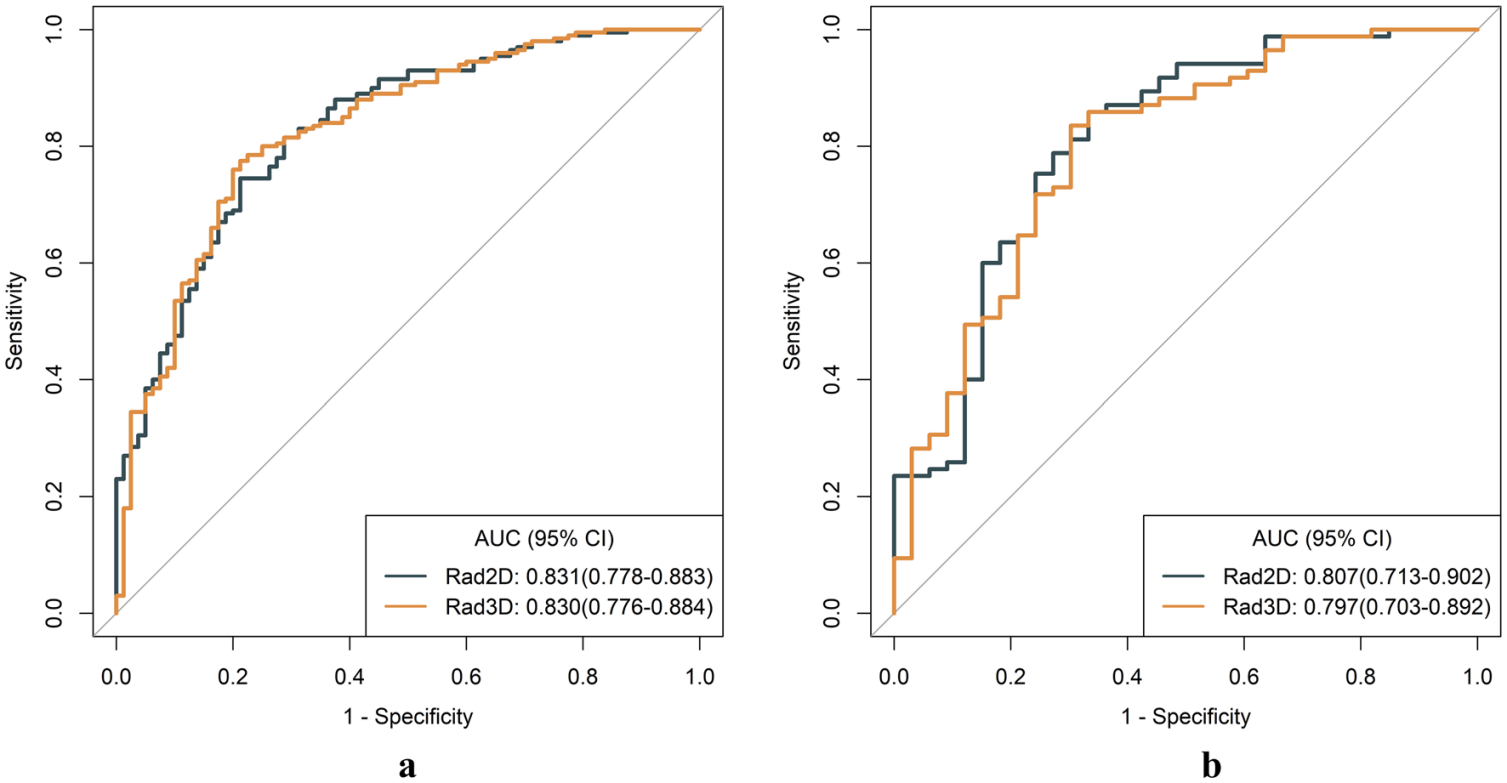
The receiver operating characteristic (ROC) curves of the 2D and 3D radiomics models. The area under the curve (AUC) values of the 2D radiomics model and the 3D radiomics model were nearly equivalent in the training (**a**) and testing (**b**) sets, and the difference between the two models was not statistically significant

**Fig. 10:**
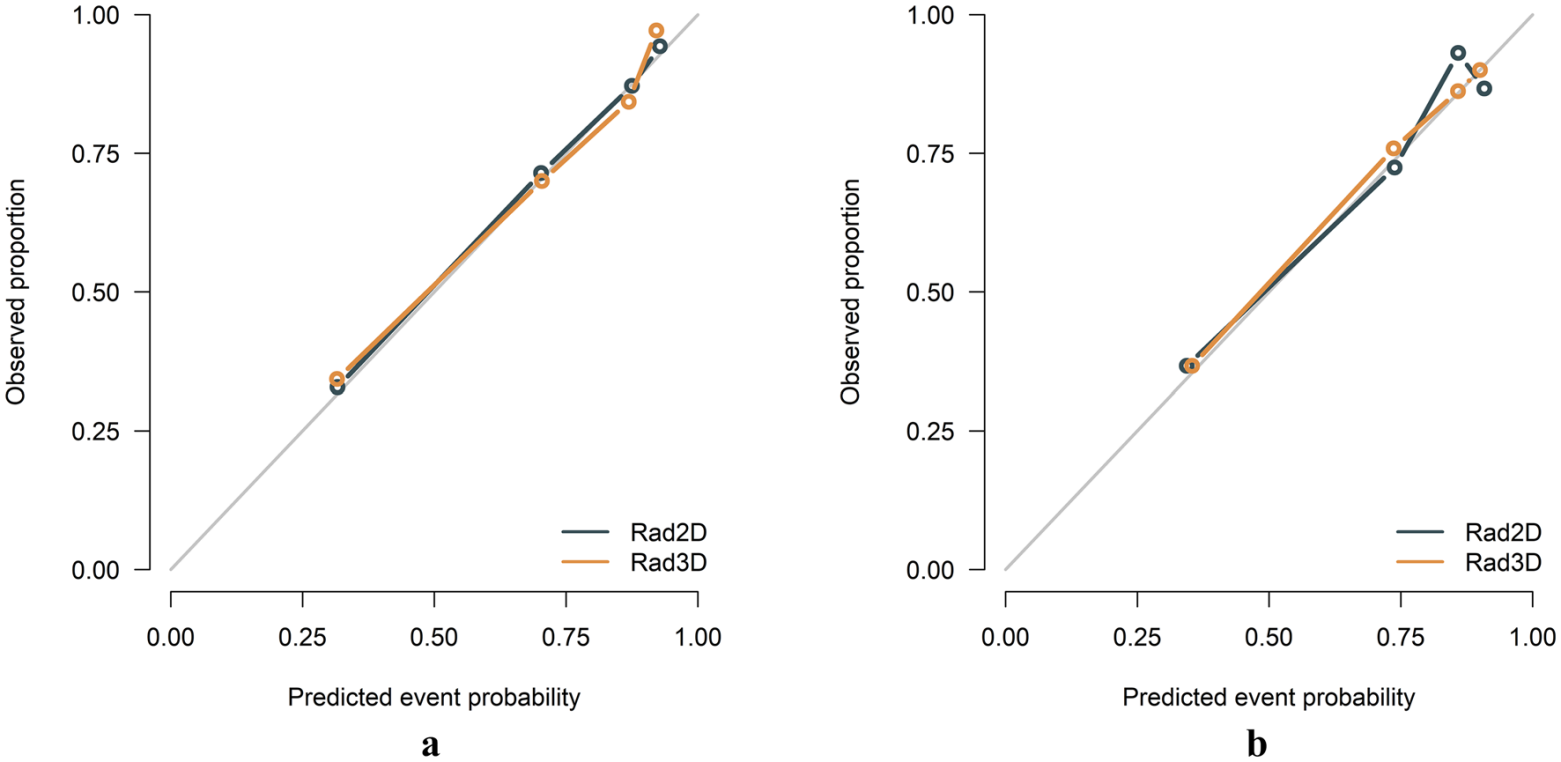
Calibration curves of the 2D and 3D radiomics models in both the training (**a**) and testing (**b**) sets. The Y-axis denoted the actual probability, whereas the X-axis signifies the predicted probability

**Fig. 11:**
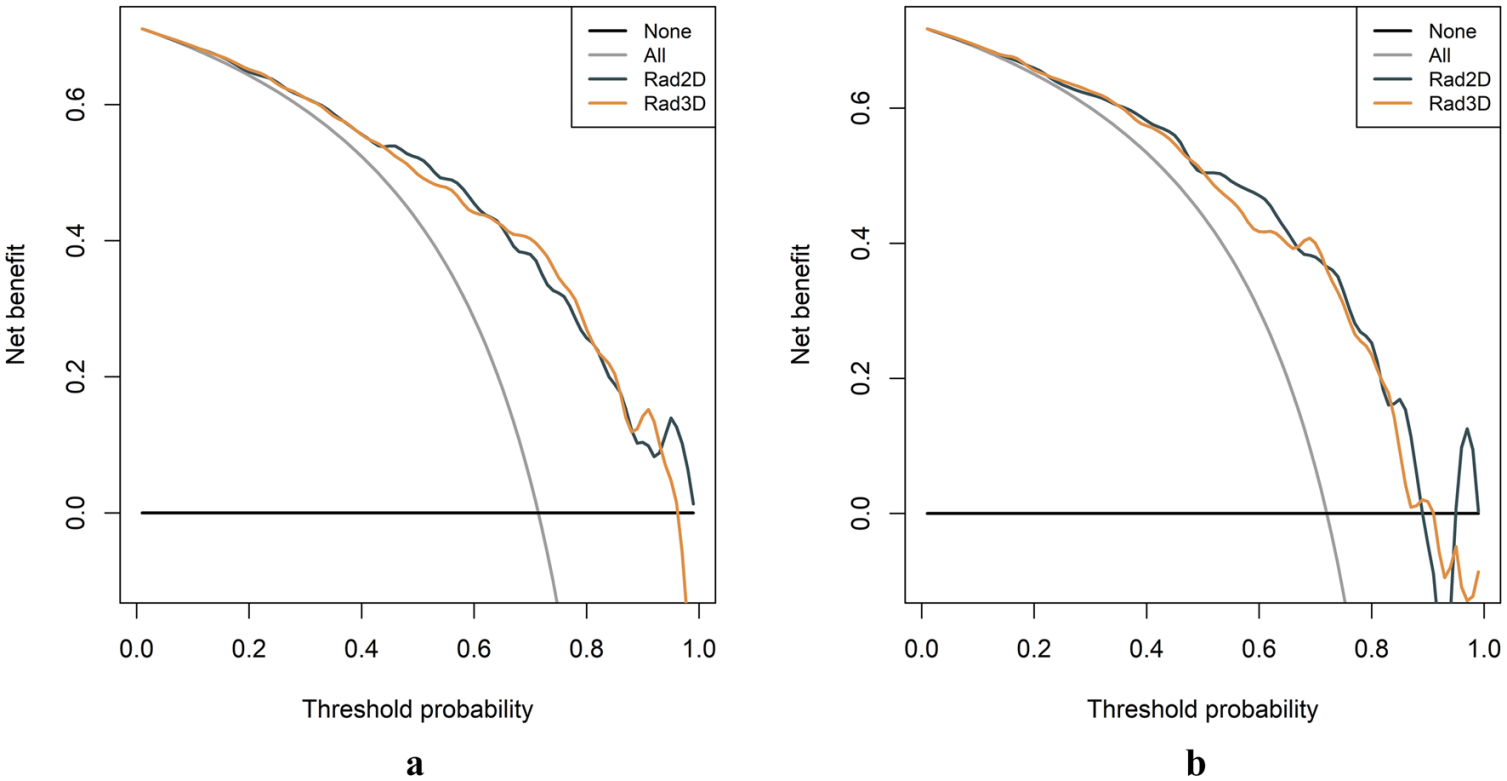
Decision curve analysis (DCA) curves were plotted for the two-dimensional (2D) and three-dimensional (3D) radiomics models in the training (**a**) and testing (**b**) sets. The black line denotes the scheme of no treatment. The dark gray line corresponds to the 2D radiomics model, whereas the yellow line represents the 3D radiomics model. DCA curves showed similar clinical utility between 2D and 3D radiomics models

Table 2 presented the detailed diagnostic performance of the models. Figure 9 depicts the ROC curves of these two models. In the training set, the AUC for 2D was 0.831 and for 3D was 0.830, with a p-value of 0.973 (DeLong test). In the testing set, the AUC for 2D was 0.807 and for 3D was 0.797, with a p-value of 0.748 (DeLong test). The 3D radiomics model had higher specificity, accuracy, and PPV in the training and testing sets. Meanwhile, the 2D radiomics model had a higher sensitivity and NPV. The calibration curves of the 2D and 3D radiomics models showed good consistency (Fig. 10). The HL test indicated no significant deviation from the perfect fit. DCA showed that the two models had a similar clinical net benefit (Fig. 11). Therefore, the performance of the 2D radiomics model in the identification of T1-2 and T3-4 stages of ESCC was comparable to that of the 3D model in terms of net clinical benefit.

**Table 2:** Diagnostic performance of two-dimensional (2D) and three-dimensional (3D) radiomics models

	AUC	ACC	SEN	SPE	PPV	NPV
Training						
2D	0.831 (0.778–0.883)	0.757 (0.703–0.806)	0.745 (0.555–0.845)	0.787 (0.650–0.863)	0.898 (0.867–0.909)	0.553 (0.505–0.575)
3D	0.830 (0.776–0.884)	0.779 (0.725–0.826)	0.775 (0.565–0.860)	0.787 (0.650–0.875)	0.901 (0.869–0.910)	0.583 (0.536–0.609)
Testing						
2D	0.807 (0.713–0.902)	0.805 (0.722–0.872)	0.871 (0.670–0.965)	0.636 (0.454–0.818)	0.860 (0.826–0.872)	0.656 (0.577–0.711)
3D	0.797 (0.703–0.892)	0.771 (0.685–0.843)	0.800 (0.482–0.918)	0.697 (0.454–0.848)	0.872 (0.804–0.886)	0.575 (0.468–0.622)

*2D* two-dimensional, *3D* three-dimensional, *AUC* area under the curve, *SEN* sensitivity, *SPE* specificity, *ACC* accuracy, *PPV* positive predictive value, *NPV* negative predictive value

## Discussion

In this study, extracted radiomics features were utilized to develop 2D and 3D radiomics models for differentiation between T1-2 and T3-4 stages of ESCC. The results showed that 2D and 3D radiomics models effectively distinguished between T1-2 and T3-4 stages of ESCC before treatment.

The CECT scan was a common diagnostic method for ESCC and was widely used in clinical practice due to its low cost, easy accessibility, and non-invasiveness [22]. However, high-order radiomics features were not incorporated into the analysis. Radiomics was a promising approach for extracting image features from radiographic images in a high-throughput manner, capable of capturing additional information that conventional images could not reveal [19]. The first step in the radiomics workflow was to acquire high-quality and standardized medical images, which were then used to segment the tumor ROI. Subsequently, high-throughput quantitative image features were extracted from the defined tumor ROI [19]. From a dimensional perspective, they could be mainly categorized into single-slice 2D ROI and whole-tumor volume 3D ROI. The 2D or 3D ROI segmentation pattern for tumors could impact radiomics feature values and the performance of discrimination models [24–26].

There had been a longstanding debate about whether 2D or 3D ROI should be used. However, the practical efficacy of radiomics features differed in clinical practice. The results were attributed to variances in image parameters, the involved organs, pathological tumor characteristics, tumor morphology, growth patterns, clinical endpoints, and the modeling methods utilized in the studies, in addition to other contributing factors. It was relatively easy for radiologists to delineate the 2D ROI of a tumor, which resulted in fewer feature calculations and less time spent [24]. Nonetheless, 3D ROI radiomics analysis covered the whole tumor volume and could better characterize the spatial heterogeneity of the tumor than 2D ROI radiomics analysis [32, 33]. However, using only a single-slice tumor ROI might not be appropriate for radiomics analysis because 2D image features could not express the heterogeneity of the whole tumor volume [34]. In our training and testing sets, the AUC values of the 2D radiomics model were 0.830 and 0.807, respectively. These values were comparable to those of the 3D radiomics model. The DeLong test did not reveal any statistically significant difference between the two models. Moreover, the DCA curved indicated that both models possess comparable clinical utility.

Currently, there is no research that compares the performance differences between 2D and 3D radiomics models in predicting T-stage for ESCC. For some solid tumors, the predictive performance of models built on 3D ROI was better or close to that of those built on 2D ROI [24, 26]. Previous studies [34–36] had demonstrated that 3D ROI contained more stable and reproducible radiomics features than 2D ROI. However, the esophagus was a hollow organ, and esophageal tumors were usually irregular. In delineating EC tumors, a specific challenge existed in determining the superior and inferior borders of the tumor. The delineation process might include varying degrees of normal esophageal tissue. Therefore, manual segmentation was necessary to ensure the accuracy of tumor ROI delineation. The issue with 2D ROI segmentation was that the selection of the maximum slice might be subjectively influenced by individual judgment, while such variability did not exist in 3D ROI segmentation. In radiomics studies of hollow organs, such as esophageal, gastric, and rectal cancers, 3D radiomics models showed superior performance compared to 2D radiomics models. Peng et al. [37] found that the 3D radiomics model had higher efficacy than 2D radiomics model in predicting the LVI status of ESCC. Huang et al. [21] found that, in gastric cancer, 3D radiomics features demonstrated greater stability and reproducibility when compared to 2D radiomics features. They also observed a higher abundance of features with an ICC > 0.75. Du et al. [38] found that the 3D radiomics model showed more encouraging performance in differentiating squamous cell carcinoma and adenocarcinoma at the gastroesophageal junction. Li et al. [39] found that 3D radiomics model outperformed 2D radiomics model in predicting LVI status in rectal cancer. Our results differed from the previous study, which was mainly due to the different pathological features considered. The largest area of the tumor could reflect the T-stage to some extent.

However, in some other studies, the performance of 2D radiomics was superior. Shen et al. [28] found that regarding the prognosis for non-small cell lung cancer, the 2D radiomics model outperformed. In another study [40], tumor heterogeneity analyzed by 2D radiomics of CT images was more accurate than 3D radiomics of tumors in predicting treatment response in patients with colorectal cancer with liver metastases. Meng et al. [25] compared the performance of 2D and 3D CT radiomics features in identifying lymph node metastasis, lymphatic invasion, and pT stage in a multicenter study of advanced gastric cancer. The results showed that the 2D model performed superiorly to the 3D model in all three tasks. Considering the time and labor required for segmentation, the 2D radiomics model was recommended for clinical applications due to its practicality and efficiency. The lower performance of the 3D model might be attributed to the additional noise introduced by the 3D ROI segmentation, which could obscure useful information and adversely affect diagnostic accuracy. Alternatively, the multi-slice segmentation used in the 3D model magnified the delineation's subjective and noise-related effects. The 3D ROI covering the whole tumor provided better average tissue property features for lesion classification in radiomics. For the delineators, obtaining a more accurate model took more time and patience, and the amount of data for analysis increased in geometric quantities. However, a potential drawback of 3D ROI was that including more segmented image slices led to more non-tumor tissue, which could distort the results [41]. The 2D ROI could minimize potential motion and respiratory artifacts to the greatest extent, and these artifacts might become more pronounced with increased ROI slices. In summary, the advantage of 2D imaging-based radiomics analysis lied in its convenience, making it the preferred segmentation method for improving efficiency without significantly sacrificing accuracy in clinical practice [42].

There were several limitations to our study that should be noted. First, as a single-center study, this led to selection bias. Furthermore, the limited number restricted the model's performance, and further external validation was necessary. Secondly, we used CT scans from two different machines to obtain the images. However, collecting data in a heterogeneous environment might improve the generalizability of our results. Third, we focused on comparing the diagnostic performance of 2D and 3D models rather than combining them with conventional CT features for diagnosis, which could be a direction for future research. In addition, another interesting direction worth exploring is to compare the differences between EUS and 2D radiomics analysis in the staging of ESCC, utilizing postoperative pathology serving as the gold standard for evaluation

In conclusion, the predictive models constructed by 2D and 3D radiomics features could be used for preoperative, non-invasive identification of T1-2 and T3-4 stages of ESCC, which had potential clinical applications for guiding individualized treatment decisions and accurately stratifying ESCC patients. In addition, 2D radiomics model may be a more feasible option due to the shorter time required for segmenting the ROI. Considering the simplicity and affordability of 2D radiomics model and its comparable predictive performance compared to 3D radiomics model, the 2D radiomics model may be a more feasible option due to the shorter time required for segmenting the ROI.

## Main points

This large retrospective study demonstrated that 2D and 3D radiomics analysis could effectively differentiate the T-stage of ESCC preoperatively.

Our results provided a new reference and direction for ESCC radiomics analysis, which could help clinicians allocate treatment.
